# Pediatric Bone Marrow Failure: A Broad Landscape in Need of Personalized Management

**DOI:** 10.3390/jcm12227185

**Published:** 2023-11-20

**Authors:** Lotte T. W. Vissers, Mirjam van der Burg, Arjan C. Lankester, Frans J. W. Smiers, Marije Bartels, Alexander B. Mohseny

**Affiliations:** 1 Laboratory for Pediatric Immunology, Department of Pediatrics, Willem-Alexander Children’s Hospital, Leiden University Medical Center, 2333 ZA Leiden, The Netherlands; l.t.w.vissers@lumc.nl (L.T.W.V.); m.van_der_burg@lumc.nl (M.v.d.B.); 2 Department of Pediatrics, Hematology and Stem Cell Transplantation, Willem-Alexander Children’s Hospital, Leiden University Medical Center, 2333 ZA Leiden, The Netherlands; a.lankester@lumc.nl (A.C.L.); f.j.smiers@lumc.nl (F.J.W.S.); 3 Department of Pediatric Hematology, Wilhelmina Children’s Hospital, University Medical Center Utrecht, 3584 CX Utrecht, The Netherlands; m.bartels-2@umcutrecht.nl

**Keywords:** cytopenia, aplastic anemia, bone marrow failure, myelodysplastic syndrome, hematopoietic stem cell transplantation, immunosuppressive therapy, gene therapy

## Abstract

Irreversible severe bone marrow failure (BMF) is a life-threatening condition in pediatric patients. Most important causes are inherited bone marrow failure syndromes (IBMFSs) and (pre)malignant diseases, such as myelodysplastic syndrome (MDS) and (idiopathic) aplastic anemia (AA). Timely treatment is essential to prevent infections and bleeding complications and increase overall survival (OS). Allogeneic hematopoietic stem cell transplantation (HSCT) provides a cure for most types of BMF but cannot restore non-hematological defects. When using a matched sibling donor (MSD) or a matched unrelated donor (MUD), the OS after HSCT ranges between 60 and 90%. Due to the introduction of post-transplantation cyclophosphamide (PT-Cy) to prevent graft versus host disease (GVHD), alternative donor HSCT can reach similar survival rates. Although HSCT can restore ineffective hematopoiesis, it is not always used as a first-line therapy due to the severe risks associated with HSCT. Therefore, depending on the underlying cause, other treatment options might be preferred. Finally, for IBMFSs with an identified genetic etiology, gene therapy might provide a novel treatment strategy as it could bypass certain limitations of HSCT. However, gene therapy for most IBMFSs is still in its infancy. This review summarizes current clinical practices for pediatric BMF, including HSCT as well as other disease-specific treatment options.

## 1. Introduction

**Figure 1:**
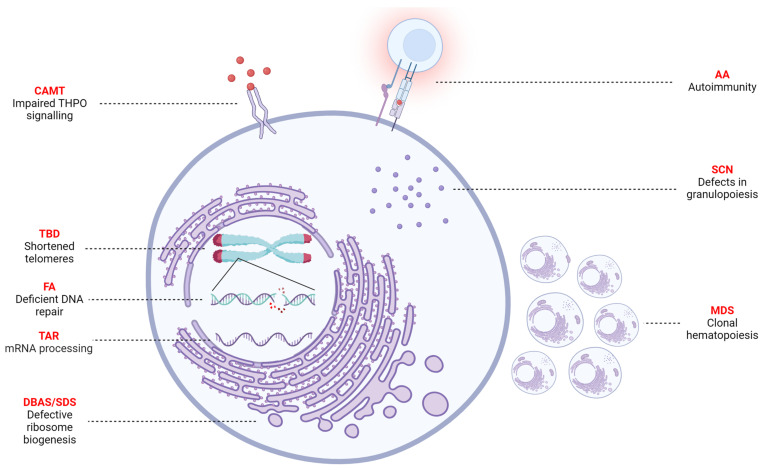
Schematic summary of the etiology of bone marrow failure (BMF). BMF is caused by a broad range of disorders, such as inherited bone marrow failure syndromes (IBMFSs), aplastic anemia (AA), and (pre)malignant diseases. Fanconi anemia (FA) is the most common IBMFS, caused by deficient DNA repair. Telomere biology disorders (TBDs) are caused by defective telomere maintenance, resulting in shortened telomeres. Diamond–Blackfan anemia syndrome (DBAS) and Schwachman–Diamond syndrome (SDS) are both caused by defects in ribosome biogenesis resulting in anemia and neutropenia, respectively. Defects in granulopoiesis can cause apoptosis of neutrophils, which is seen in severe congenital neutropenia (SCN). Congenital amegakaryocytic thrombocytopenia (CAMT) is the result of impaired THPO signaling and thrombocytopenia absent radii (TAR) is the result of aberrant mRNA processing. The main cause of AA is hypothesized to be autoimmunity. Finally, clonal hematopoiesis can result in (pre)malignant disorders, such as myelodysplastic syndrome (MDS). Figure was created using Biorender.

Peripheral (pan)cytopenia due to ineffective hematopoiesis, also defined as bone marrow failure (BMF), is a relatively frequent symptom in pediatric patients [1,2]. In most children, reversible causes such as viral infections and nutritional deficiencies underlie transient BMF [3]. On the other hand, non-reversible BMF is caused by a broad spectrum of underlying diseases, including inherited bone marrow failure syndromes (IBMFSs), malignant diseases, and (idiopathic) aplastic anemia (AA, Figure 1) [4,5,6]. In up to 50% of the patients, genetic defects are suspected as the underlying cause of non-transient BMF [7,8,9]. Next to cytopenia, IBMFS patients often show multiorgan extra-hematological defects and are at an increased risk of cancer, especially secondary myelodysplastic syndrome (MDS) and acute myeloid leukemia (AML) [4,10]. MDS results from clonal hematopoiesis and can also occur as a primary disease without the involvement of IBMFSs [11]. The remainder of patients is often diagnosed as idiopathic AA. AA is a diagnosis per exclusion, mainly comprising acquired BMF with an unknown etiology [7]. Although the exact mechanism still needs to be unraveled, the widely accepted hypothesis indicates that AA is caused by immune dysregulation [12,13].

In recent years, diagnostics of pediatric BMF have improved [7]. Timely identification of patients with non-reversible BMF is of crucial importance: it reduces the risks of invasive infections and bleeding complications and simultaneously allows for risk-adapted organ and cancer monitoring and family counseling [7,14]. In addition, it results in the prompt initiation of a treatment regime [7,15]. Although the etiology of non-transient BMF is quite distinctive, allogeneic hematological stem cell transplantation (HSCT) plays a key role in the treatment of most types of BMF [4,5,11]. This review summarizes the role of HSCT, drug therapies, as well as novel gene therapy approaches for the management of pediatric BMF.

## 2. Hematological Stem Cell Transplantation: General Aspects

Currently, allogeneic HSCT provides the only cure for most types of severe pediatric BMF. Depending on the severity of the disease and other treatment options, HSCT is provided as cellular therapy (Table 1). However, importantly, it does not correct for non-hematological features.

For pediatric BMF, the survival rate after HSCT has improved tremendously due to better donor availability, a swift initiation of HSCT, improved supportive care, and the introduction of post-transplantation cyclophosphamide (PT-Cy), currently reaching a 5-year overall survival (OS) of up to 90% [15]. In general, a matched sibling donor (MSD) is the preferred donor option. If an MSD is unavailable, matched unrelated donors (MUD) and haploidentical family donors can be considered. Alternative donor HSCT outcomes have increased remarkably since the introduction of post-transplantation cyclophosphamide (PT-Cy) treatment protocols to prevent graft versus host disease (GVHD) [16]. However, especially in IBMFS patients, the required extra PT-Cy might add to the treatment toxicity. The donor material can be collected from bone marrow (BM), umbilical cord blood (UCB), or peripheral blood stem cells (PBSCs), with the first being associated with superior survival [17]. In contrast, PBSC use is associated with higher rates of GVHD [18]. Increased risk of graft failure and delayed hematopoietic engraftment due to low cell counts present the main problems for UCB transplantation. Infusion of high cell counts and reduced intensity conditioning may help to overcome these limitations [19].

Common complications of HSCT are GVHD, alloimmunization, infections, graft failure, short- and long-term chemotherapy related organ toxicity, secondary malignancies, and transplant-related mortality [20]. Correct management can limit these complications. First of all, alloimmunization is associated with regular blood transfusions, which are often provided as supportive care before HSCT [21]. In the past, the number of blood transfusions was found to be negatively associated with engraftment [22,23]. Implementation of leukoreduction has resulted in a decreased occurrence of alloimmunization [24,25]. In addition, a swift initiation of HSCT reduced the amount of blood transfusions needed and thereby the incidence of alloimmunization. Still, in HLA-mismatched transplantation, alloimmunization can be a challenge and patients might need desensitizing treatments prior to HSCT [26]. GVHD can be managed with the use of prophylaxis, such as PT-Cy [17]. On the other hand, graft failure can be prevented by using intensified conditioning before HSCT. The exact regimen should be considered carefully for an individualized balance to achieve maximal effective bone marrow depletion while minimizing other organ toxicities [27]. As previously stated, most IBMFSs are associated with an increased risk of developing cancer [10]. Toxic conditioning regimens can further increase cancer risk and simultaneously cause other adverse effects, such as impaired growth and infertility [27]. These negative effects can be limited by using reduced intensity conditioning (RIC) regimens [27]. Lastly, age at transplantation is an important factor. Several studies have proven a younger age to be favorable for the outcome [18,28,29,30,31,32,33,34]. Therefore, HSCT should be considered as soon as possible after the confirmation of irreversible severe BMF.

## 3. Inherited Bone Marrow Failure Syndrome

### 3.1. Fanconi Anemia

Fanconi anemia (FA) is the IBMFS with the highest incidence, affecting 1–5 out of 1 million [35]. At least 22 genes are associated with FA, all with a crucial role in DNA repair [36]. In the majority of the patients, *FANCA* (64%), *FANCC* (12%), and *FANCG* (8%) disease-causing mutations are found [37]. As the products of these genes are important for detecting interstrand crosslinking of the DNA double strands and coordinating their repair through homologous recombination, these mutations can lead to unrepaired DNA double-strand breaks [38]. Therefore, FA has the highest risk for developing cancer, generally from an adolescent age with a reported cumulative risk of 15–20% at the age of 40 and 40% at the age of 50 years [10]. In addition, FA cells are hypersensitive to endogenous and exogenous stresses, leading to the unrestricted activation of the DNA damage response (hyperactive ATM/TP53/p21 pathway, ATR/CHK1 pathway, p16/RB, NF-κB, and p38) and cell cycle arrest. Thereby, oxidative stress leads to progressive TP53-dependent depletion of the hematopoietic stem and progenitor cell (HSPC) pool, ultimately leading to bone marrow failure [39]. Hematological symptoms usually start with thrombocytopenia and leukopenia and can progress towards pancytopenia [40]. The onset of severe BMF occurs mostly in childhood, with a peak in the hazard rate at 10 years of age. In early adulthood, the hazard rate decreases until the age of 30. At 50 years of age, the cumulative incidence was found to be 50% [10].

As for the expected changes in the severity of BMF and the high incidence of cancer, lifetime monitoring is crucial. Severe cytopenia, progression into MDS, or poor cytogenic abnormalities, e.g., monosomy 7, gain of chromosome 3q, complex anomalies or *RUNX1* abnormalities, are all indications for HSCT [41,42]. The outcome of HSCT has increased remarkably due to the introduction of fludarabine (Flu) in combination with T-cell depletion. Flu-based RIC has been associated with improved survival and lower rates of GVHD [43,44,45,46]. The main goal of T-cell depletion is to decrease GVHD [47]. GVHD, next to causing increased transplant-related mortality, has been associated with the development of post-transplant malignancies [44,48,49]. Post-transplant malignancies are seen in many patients. After 20 years post-HSCT, the overall cumulative risk of cancer was 34% [44]. Thus, GVHD should be limited, thereby limiting the incidence of malignancies. Both in vivo and ex vivo, T-cell depletion could prevent GVHD [47]. In vivo depletion relies mainly on post-HSCT administration of cyclophosphamide (Cy) [47].

Two large studies with MSD HSCT using Flu- or Cy-based conditioning regimens reported an OS of 81% and 76% [44,50]. Using a conditioning regimen consisting of Flu with Cy, Benajiba et al. reported a 2-year OS of 95% [51]. Similarly, Bonfim et al. reported a 5-year OS of 95% using Cy with or without rabbit anti-thymocyte globulin (ATG) in matched related donors [52]. Finally, 85% of patients treated with Flu and Cy conditioning in combination with alemtuzumab, administered as a T-cell depletion regimen, were alive after five years. Alemtuzumab was associated with increased OS [53]. Concludingly, nonirradiated Flu- and/or Cy-based conditioning regimens for MSD HSCT provide excellent survival rates.

The outcome for MUD and mismatched donor HSCT has improved over the years after the implementation of PT-Cy [17,43,47,52,53,54,55]. The use of haploidentical donors in FA patients also showed promising results, with an OS ranging between 73% and 100% [52,56,57,58,59]. These high OS rates are mainly achieved due to the implementation of ex vivo or in vivo T-cell depletion with PT-Cy, reducing the development of GVHD. HSCT becomes less favorable if progression into MDS or AML has occurred. A 5-year OS of just 42–55% has been reported for patients with progression into MDS or AML [60,61]. AML is an especially poor prognostic factor, with an OS of 44% compared to an OS of 71% and 89% for FA patients with MDS and without hematological malignancies, respectively [62].

Androgen therapy could improve blood counts and 68% of the patients were reported to respond to androgens [63]. However, pre-HSCT androgen administration is associated with higher GVHD rates and decreased OS [46,64]. In addition, high rates of hepatic adenomas are observed in patients treated with androgens [63,65,66]. Thus, androgens should only be used for patients with severe symptoms that are not eligible for HSCT.

### 3.2. Telomere Biology Disorders (TBDs)

Recent developments in molecular analysis and telomere length measurement have identified a broad spectrum of telomere biology disorders (TBDs) beyond the classic dyskeratosis congenita (DC). TBDs are caused by a spectrum of germline mutations resulting in defective telomere maintenance [67,68]. As a consequence, shortened telomeres, which limit cell proliferation and cell survival, emerge [67,68]. Not all patients develop the classic and more severe DC symptoms, with the triad of nail dysplasia, abnormal skin pigmentation, oral leukoplakia occurring with BMF at a young age [69]. Revesz syndrome, Hoyeraal–Hreidarsson syndrome, and Coats plus are severe subtypes that emerge in early childhood and are accompanied by complex multisystem disorders [67]. In contrast, other patients can remain asymptomatic for extended periods [67,69]. Strikingly, the heterogeneity of TBD can even be observed within the same family and genetic anticipation is described [67]. Other TBD-related risks include cancer as well as pulmonary and liver dysfunction [67]. BMF, which eventually develops in 80 to 90% of patients, is the most common cause of death [70].

Drug-based treatment for BMF consists of androgen therapy, and is mainly used for the treatment of cytopenia and DC-associated pulmonary fibrosis in adult patients. However, recent trials investigated broader application of androgens in TBDs including pediatric patients [71]. Although hematological defects can respond to androgens, the only current curative treatment for these patients is HSCT [72]. Toxic effects such as virilization are frequently reported with androgen treatment [73]. Currently, danazol, which is less toxic and virilizing, is the most widely used medicine for DC [74]. The exact working mechanisms of danazol and other synthetic androgens are yet to be determined. There are currently two hypotheses: First, it is thought that androgens could elicit a hematological effect via estrogen-mediated telomerase activity [75]. However, it seems that danazol cannot be aromatized into estrogen, which would be required for the hypothesis to be true [76]. Secondly, androgens might result in erythropoiesis by acting on the erythropoietin (EPO) receptor [77]. In agreement with this hypothesis, Townsley et al. reported telomere elongation after danazol treatment, hypothetically by the hormone-mediated upregulation of *TERT* and of telomerase enzymatic activity [78]. A positive correlation between testosterone metabolites and telomere length was found [79]. However, Khincha et al. found no significant difference in telomere length between androgen-treated and untreated DC patients [76]. In addition, no elongation was observed after the treatment with the synthetic androgen oxymetholone [80]. Lastly, androgen deprivation therapy did not influence telomere length in patients with prostate cancer [81]. Thus, the exact working mechanisms of androgen therapy and its effect on telomere length remain to be elucidated.

The potentially curative treatment for severe BMF in TBD is HSCT. HSCT data for pediatric DC is scarce and often combined with the data of adult patients. In a literature review including patients of all ages, Alter et al. reported a 5-year OS of 71% for MSD HSCT, whereas only 31% of the patients receiving HSCT from alternative donors were alive after two years [82]. Similarly, Fioredda et al. found a 3-year OS of 73% and 46% for HLA-matched versus mismatched HSCT, respectively [33]. Unfortunately, long-term outcomes are disappointing, mainly due to unacceptable transplant-related toxicities. Common causes of death after HSCT are infections, pulmonary disease, and secondary cancers, resulting in a long-term OS of less than 30% [82,83,84]. The use of RIC regimens might reduce transplant-related toxicities. However, the rarity of the disease in combination with the wide variety of used treatment protocols limit the understanding of late complications in transplanted IBMFS patients. The reported OS after using RIC is 67%, with an exceptionally high OS of 60% for patients transplanted using unrelated donor sources [85]. In contrast, RIC did not significantly improve survival in a large systematic review containing 109 patients. However, the occurrence of pulmonary diseases seemed slightly lower after using RIC regimens [83]. Therefore, RIC regimens might improve OS in the long run by limiting the progression of pulmonary diseases or other transplant-related toxicities [86].

Post-HSCT management of DC patients remains challenging due to the many comorbidities that they suffer from. There is a need for novel and better treatment options. The granulocyte colony-stimulating factor (G-CSF) can induce a temporary effect but is limiting as it cannot be administered in combination with androgen therapy due to a high risk of splenic peliosis and rupture [70,87]. Recently, PAPD5 inhibitors have been developed that could restore telomere length [88,89]. PAPD5 inhibitors might be beneficial for DC patients with reduced TERC levels. In addition, thymidine (dT) supplementation and *SAMHD1* inhibition were able to induce telomere synthesis in induced pluripotent stem cells (iPSCs) derived from TBD patients [90]. However, no clinical trials have started yet.

### 3.3. Diamond–Blackfan Anemia Syndrome

Defects in ribosome biogenesis are the underlying cause of Diamond–Blackfan anemia syndrome (DBAS). In the majority of cases, mutations in genes encoding for ribosome proteins (RP) are found, of which *RPS19* is most frequently affected (25% of all cases) [91]. The pathogenesis of defective rRNA maturation and the erythroid defect in DBAS is not fully defined. However, the stabilization of p53 and the activation of p53 targets are responsible for the activation of apoptosis, and the cell cycle is most often hypothesized [92]. Recent studies have identified a role for specifically decreased GATA1 mRNA translation and excess free heme-induced apoptosis in the DBAS erythroid phenotype [93,94]. As a consequence, erythroid cells undergo cell cycle arrest and apoptosis, resulting in (severe) anemia [95]. In addition to hypoplastic anemia, approximately 50% of patients have congenital malformations, including skeletal abnormalities and cardiac defects [96,97]. In line with the other inherited bone marrow failure disorders, DBAS is associated with an increased cancer risk [98,99].

Treatment options for DBAS patients are partially age-dependent. For patients older than one year and suffering from severe anemia (Hb < 8 g/dL), glucocorticoids (usually prednisone) can be used to stimulate erythroid expansion and alleviate anemia. Consequently, up to 80% of the patients initially respond, and responses can decline over time with the doses needing to be tapered to acceptable dose proportions for chronic treatment (max 0.3 mg/kg/day) [92,97,100,101]. Since glucocorticoid treatment is generally associated with significant toxicity and negatively affects growth and neurocognitive development, it is specifically not recommended in patients below the age of one [101,102]. As a result, infants are treated with chronic blood transfusions [103]. Chelation therapy is essential in all patients treated with chronic transfusions to prevent a severe iron overload, which generally begins occurring after >10 transfusions. This in agreement with consensus guidelines [101]. Interestingly, independent of prior treatment and genotype, some DBAS patients can enter remission, which is defined as an adequate hemoglobin level without the need of treatment lasting for at least six months [100,104]. How these patients enter remission is still largely unknown.

Patients become eligible for HSCT if they remain transfusion-dependent, with or without an iron overload, require toxic levels of steroids, or, although rare at a pediatric age, develop MDS/AML [17]. The OS of DBAS patients treated with HSCT has increased tremendously from 29% to 91% [29,30,31,97,105]. Moreover, earlier studies reported a lower OS for MUD HSCT, and the reported OS is currently comparable for MSD and MUD HSCT [29,30,84,105]. Due to this development, HSCT could even be considered a first-line treatment option when an MSD or MUD is available. Thereby, the negative effects of steroid treatment and blood transfusions, such as growth deficiencies and iron toxicity, are prevented, and a long-term cure is simultaneously provided. The data on alternative donor sources is limited. Darrigo Junior et al. reported an OS of 29% for patients receiving an HLA-mismatched HSCT and therefore recommended to avoid HLA-mismatched HSCT whenever possible [32]. However, just seven patients were included, of which only one received PT-Cy. In addition, unrelated umbilical cord blood transplantation (UCBT) was found to be unfavorable [32]. In contrast, two other studies did not find UCBT to be inferior [29,31]. As only alternative donor sources, such as HLA-mismatched HSCT and UCBT, have been used in a limited number of DBAS patients, it is difficult to advocate recommendations. Therefore, more research is essential.

Although not as high as other IBMFSs, DBAS patients have an increased risk of developing malignancies. Solid tumors, in specific osteogenic sarcoma and colon carcinoma, were more often reported in addition to hematological malignancies [97,106]. Despite limited evidence, the type and incidence of malignancy seems to be dependent on the underlying mutation as well. For example, *RPS26* mutations have not been associated with MDS or cancer thus far [99]. DBAS patients are mainly treated with myeloablative conditioning (MAC), typically Busulfan (Bu) or Treosulfan (Treo)-based medications [29]. Treo has been associated with lower rates of liver toxicity and infertility, making it a more favorable treatment option [31,107]. Although rarely used, preliminary results suggest that Flu-based RIC regimens are also safe and effective in DBAS [30,108].

Recent studies have unveiled emerging treatment strategies for DBAS. Metformin [109], Trifluoperazine (TFP) [110,111], Sotatercept [112], and L-leucine [113,114] have shown efficacy in improving symptoms in preclinical DBAS models. For TFP (NCT03966053), Sotatercept (NCT01464164), L-leucine (NCT01362595), and NCT02386267), clinical trials are being conducted.

### 3.4. Shwachman–Diamond Syndrome

Similar to DBAS, defective ribosome biogenesis and maturation underlies the Shwachman–Diamond syndrome (SDS). However, whereas red blood cells are the most affected in DBAS, SDS is characterized by neutropenia [115]. In addition, patients might develop anemia and thrombocytopenia as well as other extra-hematological multiorgan defects, such as exocrine pancreatic dysfunction [116]. Around 90% of patients have mutations in the *SBDS* gene [117]. Other genes that cause an SDS(-like) phenotype include *DNAJC21*, *EFL1*, and *SRP54*. Interestingly, all genes engage in ribosome biogenesis: SBDS, through a direct interaction with EFL1, promotes the release of the eukaryotic initiation factor 6 (eIF6) during ribosome maturation; DNAJC21 stabilizes the 80S ribosome; and SRP54 facilitates protein trafficking. These recently identified genes underline the postulate that SDS is a ribosomopathy [118].

In general, SDS patients develop mild to moderate neutropenia, and treatment is not necessary. G-CSF can be administered to patients with recurrent infections and/or severe neutropenia. HSCT is only contemplated when patients with severe neutropenia are unresponsive to G-CSF or if progression to MDS or AML occurs [116]. The reported OS of HSCT is around 60 to 65% [119,120,121,122]. However, a significant discrepancy is seen between those transplanted for BMF and those treated for secondary MDS or AML, with a reported OS of around 70% compared to 15–30%, respectively [119,122]. Progression into AML is especially associated with a poor prognosis, with an OS between 11 and 19% as compared to an OS of 51 to 56% for SDS patients with secondary MDS [120,123]. OS of secondary MDS or AML is low due to high relapse rates and transplant-related toxicity [119]. Therefore, it is essential to initiate HSCT before its progressing into MDS or AML.

However, as only a small percentage of SDS patients eventually develops severe cytopenia or malignancies, preemptive transplantation is not recommended [124,125]. Although high-dose G-CSF treatment is correlated with an increased risk of MDS or AML; recent studies show that clonal evolution is controlled by several factors, including the disease causing germline mutation, additional somatic mutations especially *RUNX-1* and biallelic *TP53* variants, cellular stressors, and stromal inflammation [126,127]. Therefore, an effective surveillance strategy is challenging yet necessary, which might require regular bone marrow aspirate and biopsy screening, which also includes screening for additional somatic mutations and clonal evolution [125]. The surveillance strategy should also underline that clonal evolution is not always equal to a malignant transformation and that it even might be part of a rescue mechanism leading to improvements in BMF and blood counts [128]. The need for sufficient surveillance was emphasized by Myers et al., who reported a 3-year OS of 62% for patients with surveillance compared to a low OS of 28% for those without [123]. However, due to its retrospective nature, there might be bias within this study. Patients undergoing surveillance might also be more compliant with other care and therefore have higher survival rates.

The RIC regimen can be safely used in SDS patients [129]. As no differences between the RIC and MAC regimens have been observed yet, RIC regimens are recommended [119,122].

### 3.5. Severe Congenital Neutropenia

Severe congenital neutropenia (SCN), previously known as Kostmann’s syndrome, is characterized by severe neutropenia, and a specific neutrophil maturation defect at the (pro)myelocyte stage of neutrophil development. About half of the patients have a heterozygous *ELANE* mutation encoding for neutrophil elastase [130,131]. Further, the pathophysiology has not been completely elucidated, it has been shown that apoptosis of neutrophils is induced as a result of misfolded proteins [130,132]. Other genes involved in SCN include *HAX1*, *WAS*, *JAGN1*, *VSP45*, *GFI1b*, and *G6PC3* [131,132]. Although there is heterogeneity in the etiology, increased apoptosis of neutrophils is seen in these SCN subtypes [132]. Unlike many other IBMFSs, patients with *ELANE* mutations often display no or limited extra-hematological features [132]. In contrast, extra-hematological defects can be seen in other patients with other genetic causes [133].

G-CSF, usually filgrastim, is offered as treatment in patients with severe neutropenia (ANC < 0.5 × 10^9^/L), which is seen in the majority of patients. Additionally, pegfilgrastim, the pegylated form of filgrastim, might be suitable for patients with poor compliance or side effects of classical G-CSF [134]. SCN patients might also benefit from the addition of nicotinamide (vitamin B3) to G-CSF treatment; however, the proven additive value of nicotinamide is yet to be investigated [135]. Of those treated with G-CSF, 90% have adequate responses [136]. Thereby, life-threatening sepsis is prevented. HSCT is indicated in patients who have a low or absent response or require high doses of G-CSF. An absent, low, or decreasing response to GCSF might also suggest the presence of *CSF3R* mutations, which are associated with a higher risk of progression into secondary myeloid malignancy [137]. However, the presence of *CSF3R* variants is not sufficient for the prediction of myeloid malignancy in SCN patients. Recent studies show that *CSF3R*-mutant clonal hematopoiesis can persist for many years without malignant progression. Additional somatic loss-of-function variants of *RUNX1* may be an early progression event, followed by the acquisition of chromosomal abnormalities (e.g., monosomy 7 or trisomy 21) [126]. Progression into MDS or AML is reported in two independent long-term studies, which indicate a cumulative risk of 22% and 11%, respectively [137,138]. Recent reports show excellent HSCT outcomes for patients with SCN with (OS 79–83%) or without (78–87%) malignancy at the time of transplant [18,126,139]. Interestingly, in these studies, the malignant relapse rate after HSCT in SCN patients with an active myeloid malignancy at the time of transplantation is very low: this might be explained by reduced fitness of SCN leukemic stem cells. Comparable HSCT outcomes in SCN patients with or without a malignancy support a wait-and-see approach in SCN patients who are responsive to G-CSF.

### 3.6. Congenital Amegakaryocytic Thrombocytopenia

Congenital amegakaryocytic thrombocytopenia (CAMT) is characterized by ineffective megakaryopoiesis and causes severe thrombocytopenia at birth which gradually progresses into pancytopenia during the first years of life [140,141]. In the majority of cases, CAMT is caused by mutations in the *MPL* gene encoding for the thrombopoietin (THPO) receptor [141]. However, mutations in the *THPO* gene itself and defective regulation of MPL signaling have also been reported [142,143,144]. CAMT, in association with radio-ulnar synostosis (RUSAT), is mostly caused by mutations in *MECOM* and rarely in *HOXA11*.

In general, HSCT is seen as the golden standard for curing BMF in CAMT patients. Two extensive retrospective studies have been performed, reporting a 5-year OS of 77% and 86% after HSCT [28,145]. For patients receiving HLA-matched HSCT, the 5-year OS is significantly higher as compared to HLA-mismatched transplantations (93% and 75%, respectively; *p* = 0.04) [28]. Although not significant, the researchers also found differences in 5-year graft failure-free survival between both groups (87% and 75%, respectively) [28]. In a small study, Mahadeo et al. found an exceptionally high OS of 100% for patients treated with HLA-mismatched and unrelated UCBT [146]. In addition, Bizzetto et al. reported promising results for unrelated UCBT, with 11 out of 13 patients being alive at the end of the study [84]. These results indicate HLA-mismatched and unrelated UCBT to be a suitable treatment strategy when MSD HSCT is unavailable. MAC is most commonly used as a conditioning regimen for CAMT patients. In CAMT, RIC regimens have rarely been used [28,147]. Although only used in a few cases, Woods et al. stated that the use of RIC regimens might also be beneficial for CAMT [147]. The actual practical effects of RIC regimens in CAMT still need to be confirmed.

Although HSCT is considered as a curative treatment for CAMT, not all patients will respond. Seo et al. showed that a *THPO* mutation could not be cured with HSCT due to it being an extrinsic factor of hematopoiesis [148]. Instead, romiplostim, a THPO receptor agonist, seems to provide sufficient treatment; however, long-term data is still lacking [148,149]. These results emphasize the need for genetic diagnostic screening to provide the correct treatment regimen.

### 3.7. Thrombocytopenia Absent Radii

Thrombocytopenia absent radii (TAR) is caused by a null allele in combination with a hypomorphic allele for the *RBM8A* gene, which normally encodes for proteins involved in mRNA processing. Abnormal transcription of *RBM8A* results in bilateral absent radii and thrombocytopenia [150,151]. Although it never reaches normal levels, the thrombocytopenia usually stabilizes after two years [152]. Therefore, HSCT is not recommended. Instead, platelet transfusions are generally used. In addition, platelet-stimulating agents such as romiplostim and oprelvekin have shown promising results [152,153]. In rare cases, secondary MDS or AML has been reported [154]. However, it is unknown whether TAR, like many other IBMFSs, is associated with an increased risk for secondary malignancies.

## 4. (Idiopathic) Aplastic Anemia

In pediatric patients, AA is an umbrella term for the remaining patients with peripheral cytopenia and hypoplastic bone marrow with an unidentified (genetic) cause despite extensive diagnostics [7]. AA is hypothesized to be driven by an immunologic etiology. The strongest evidence for an immune mechanism in these patients is the result of immunosuppressive therapy (IST) restoring blood counts in a part of AA patients [155]. In addition, the role of immune dysregulation is supported by the identification of oligoclonal expanded T-cell populations in experimental settings [156]. Extensive experimental investigations have provided data supportive for an immune-mediated pathophysiology. Several related mechanisms have been suggested, including CD8^+^CD57^+^ oligoclonal T-cells with a direct cytotoxic activity [12], secretion of different inflammatory cytokines such as interferon-γ (IFN-γ) [157], immune disarrangement by increased T-helper type 17 cells [158] or reduced regulatory T cells (Tregs) [159], and associative correlations with certain HLA types [160].

### 4.1. Paroxysmal Nocturnal Hematuria

Paroxysmal nocturnal hematuria (PNH) is clinically associated with intravascular hemolysis, nocturnal hemoglobinuria, thrombosis, and bone marrow failure. Although frequently found in adult AA patients, PNH is rarely associated with bone marrow failure in pediatric AA. PNH is caused by a somatic mutation in the phosphatidylinositol glycan anchor biosynthesis, class A (*PIG-A*) gene in the hematopoietic stem cell, rendering cells susceptible to complement-mediated hemolysis. Pediatric patients with AA often have small PNH clones, but do not display the characteristic clinical phenotype of PNH. Clone size is an important predictor of clinical symptoms, especially considering the risk for thrombosis. Patients with small PNH clones should be followed-up at regular intervals for potential progression/evolution of these clones, and/or development of the clinical phenotype of PNH [161]. Differentiation from PNH requires peripheral blood flowcytometry for glycophosphoinositol-linked cell surface membrane proteins (CD55/CD59). Treatment of PNH was revolutionized with the advent of complement binding antibodies, such as eculizumab, ravulizumab, and pegcetacoplan [162,163,164].

### 4.2. Treatment

For the management of AA, optimal supportive care is crucial until curative treatment is successfully applied as the complication risks of cytopenia are high [21]. Supportive care includes red blood cell and platelet transfusions as well as antibiotic and antifungal effective prophylaxis. In patients with severe infections, G-CSF might be used, and granulocyte transfusions might be considered.

In contrast to adult patients, the use of IST in pediatric severe AA patients is limited to those cases where HSCT is not feasible due to the lack of a suitable donor, patients’ condition, or no access to an HSCT center. The main argument against IST in pediatric patients suitable for HSCT is the inferior response rate (40–60%) with a significantly lower OS of patients undergoing salvage HSCT after a failed IST as compared to upfront HSCT [165,166]. Clinically applicable diagnostic tests to identify patients with immune AA and/or markers to predict the IST response are needed to broaden the use of IST in pediatric AA patients. If IST is considered, recent studies prove there is a significant improvement in the treatment response by adding Eltrombopag to the standard regiments of horse ATG combined with CsA. In addition, IST with Eltrombopag is an upcoming therapy for less severe SAA patients [167,168].

In pediatric and AA patients below 40 years of age, HSCT with an MSD is the upfront treatment modality [14]. HSCT with MSD has reached an OS of 90% [169,170,171]. In general, bone marrow is recommended as the donor source since PBSCs are associated with a higher risk of GVHD and mortality [172,173]. Based on a comparison of several conditioning regimens, most pediatric SAA patients receive reduced-intensity conditioning regimens, mainly Flu in combination with Cy and ATG independent of the type of donor [174].

In recent years, the use of alternative donors and donor sources have shown promising results. Bacigalupo et al. noticed that the usage of MUD is not significantly inferior to MSD [175]. Similarly, Dufour et al. showed that upfront MUD is similar to MSD. In addition, although OS between MUD HSCT and IST is similar, the 2-year event-free survival of MUD HSCT was significantly higher (92% and 40%, respectively) [165]. Furthermore, HSCT with haploidentical donors showed OS ranging between 65 and 85% [176,177,178,179]. Xu et al. even showed a haploidentical donor transplantation to be suitable as an upfront therapy with OS, and showed event-free survival to be similar to matched related donors [180]. Lastly, UCBT did show promising results with a 5-year OS of 94% when transplants took place after 2006 [181]. Based on these results, alternative donors and donor sources should be considered as an upfront therapy for pediatric AA patients if MSD is unavailable. This results in a change of the treatment algorithm, with MSD, MUD, mismatched HSCT, and IST being preferred in this sequential order.

## 5. Syndromes with a Malignant Predisposition

### 5.1. Myelodysplastic Syndrome

MDS can occur as a secondary disease to IBMFSs and AA. However, it can also occur as a primary disease. In contrast to the elderly patients, primary MDS is a rare disorder in children [11,182]. MDS patients often exhibit pancytopenia and hypocellular BM [182]. Based on the information from the World Health Organization (WHO), childhood MDS can be further subdivided depending on the amount of blast in the peripheral blood (PB) and BM: Childhood MDS with low blasts (cMDS-LB), formally known as refractory cytopenia of childhood (RCC), is defined as less than 2% blast in the PB and less than 5% in the BM. If the blasts exceed this percentage but stay below 20% in both the PB and BM, it is known as childhood MDS with increased blasts (cMDS-IB). A blast percentage above 20% is seen as AML [183]. However, be aware that a diagnosis should not be solely based on the blast percentage in a single specimen.

Patients with cMDS-LB can remain stable for an extended period of time without transfusion dependence, severe cytopenia, or infections. Therefore, often a wait-and-see strategy is used for these patients [11,182]. If the disease progresses or when certain risk markers, such as monosomy 7, are present, HSCT is advised. Patients with monosomy 7 have a higher probability of their disease progressing into cMDS-IB and AML; however, monosomy 7 can also display itself as part of a rescue mechanism for an underlying constitutional disorder with pathogenic variants in the genes for *GATA2* or *SAMD9/SAMD9L* [184,185]. Progression into cMDS-IB should be avoided at all costs since this is associated with worse survival [184]. In the past, MAC regimens were used, but high incidences of transplantation-related mortalities were seen [11,186]. Instead, RIC regimens can be used safely for cMDS-LB patients [187]. The latest reports showed an OS of 94% and an EFS of 88% [188]. Thus, RIC regimens are promising in reducing toxicity-related death, thereby improving OS for cMDS-LB patients.

For cMDS-IB, consensus about the preferable conditioning regimen is lacking. The European Working Group on pediatric MDS (EWOG-MDS) recommends using MAC regimens before HSCT [189]. Treatment with MAC resulted in a 5-year OS and probability of EFS of 63% [190]. However, especially for IBMFS patients, RIC might be preferred due to decreased transplant-related toxicity. Preliminary data suggest RIC regimens to be associated with a high incidence of relapse [189]. Therefore, if RIC is chosen, it is crucial to rigorously monitor the risk of relapse and integrate early preemptive treatment measures. In addition, the use of intensive chemotherapy before HSCT remains controversial. Moreover, it could induce a reduction of blasts, and severe toxicity is also associated with chemotherapy. In general, the EWOG-MDS does not recommend intensive chemotherapy for cMDS-IB [190]. Again, no consensus has been reached yet [189].

### 5.2. Other Germline Predisposition for MDS/AML

Germline mutations, such as *GATA2*, *SAMD9/9L*, *RUNX1*, *CEBPA*, and *ETV6*, are increasingly recognized as predisposing factors to myeloid neoplasms, such as MDS. In children, GATA2 and SAMD9/9L syndromes are the most prevalent predisposing conditions causing primary MDS. *GATA2* is a transcription factor vital for multilineage hematopoiesis and its deficiency due to loss of function (LOF) mutations can cause a variability of clinical symptoms, including hematological and extra-hematological abnormalities, including immunodeficiency, lymphoedema, and alveolar proteinosis. The disorder is highly penetrant as around 75% of the patients will develop a form of myeloid neoplasm [185].

*SAMD9/9L* mutations are located on chromosome 7 and function as regulators of cell proliferation. *SAMD9* gain of function (GOF) mutations cause a combination of myelodysplasia, infections, restriction of growth, adrenal hypoplasia, genital phenotypes, and enteropathy, which is known as the MIRAGE syndrome. *SAMD9L* GOF mutations are associated with ataxia-pancytopenia (ATXPC), which is characterized by neurologic symptoms in combination with pancytopenia [191,192]. Both *GATA2* and *SAMD9/9L* are linked to the emergence of monosomy 7 and are together responsible for at least 50% of pediatric MDS with monosomy 7 [185].

Clonal hematopoiesis, such as monosomy 7, is often seen as a risk factor for myeloid neoplasms. However, increasing evidence is available that clonal hematopoiesis does not equate to a malignant transformation by itself [191]. This can, for example, be seen in *SAMD9/9L* mutation-driven BMF. In these patients, genetic reversion is not uncommon. During this process, hematopoietic cells acquire additional somatic mutation that help resolve the pancytopenia. Up until now, three different mechanisms of genetic reversion have been identified [192]. First, loss of heterozygosity of chromosome 7q with uniparental disomy (UPD) could result in the loss of the mutant allele [192,193]. Second, in cis LOF mutations could counteract the GOF mutations and cause a recovery of the cytopenia. Finally, the mutant allele can be eliminated via a (partial) loss of chromosome 7, a process referred to as an adaptation with aneuploidy. Consequently, the first two mechanisms can rectify the cytopenia, and aberrations of chromosome 7 have been associated with a progression towards MDS in multiple patients [194,195]. Interestingly, for both *SAMD9* and *SAMD9L*, multiple clones with different mechanisms of reversion have been found within the same patient [195,196]. Thus, it seems that the cells are under selective pressure to revert the GOF mutations.

As these findings are relatively new, no clear evidence-based conclusion regarding a treatment strategy can be formed yet. As monosomy 7 has been identified as a risk marker for its progression into cMDS-IB, preemptive HSCT is often advised. However, this strategy might not be required for patients undergoing genetic reversion. Genetic reversion via UPD or in cis LOF mutations seem to be part of an escape mechanism to restore hematopoiesis and should therefore not require immediate definitive treatment to prevent a malignant transformation.

## 6. Gene Therapy as a Novel Treatment Strategy

For BMF disorders with an identifiable monogenetic etiology, gene therapy might prove to be a new cure to correct ineffective hematopoiesis while circumventing certain limitations of HSCT, such as donor restriction and GVHD [197]. In general, gene therapy is based on two different strategies [198]. First of all, viruses can be exploited to incorporate genes into the DNA [198,199]. Most commonly, lentiviruses are used as they can safely integrate genes in both dividing and nondividing cells [199]. Although lentiviral vectors are associated with excellent safety, the risk of cancer remains due to insertional oncogenesis [197]. In contrast, gene editing techniques are able to modify genes. Most commonly, the clustered regularly interspaced short palindromic repeat and CRISPR-associated protein 9 (CRISPR/Cas9) are used, which induce a site-specific double-strand break (DSB). DSB can be repaired with non-homologous end joining (NHEJ) or homology-directed repair (HDR). NHEJ is error-prone, resulting in insertions or deletions around the break. In contrast, HDR uses homologous repair. HDR can be used to replace a sequence or to insert a novel gene segment [198,200]. CRISPR/Cas9 is not entirely safe yet, as it can cause off-target effects and DNA rearrangements [197,201].

Gene therapy for IBMFSs is still in its infancy. For FA patients, gene therapy has mostly been focused on correcting *FANCA* mutations. Lentiviral-mediated *FANCA* transduction has already been assessed in phase I/II clinical trials, and (preliminary) data have been promising, with all patients achieving stable blood counts [202,203]. Although several studies provided evidence that HDR can correct FA mutations, it might not be the most appropriate technique since HDR is partially hampered in FA patients [204,205,206]. Instead, FA cells make more frequent use of NHEJ [206]. Román-Rodríguez et al. found that NHEJ could be used to restore frameshift mutations in hematopoietic stem cells from FA patients [207]. However, the technique is limited to specific missense and small frameshift mutations.

For DC, a clinical trial is now ongoing (NCT04211714), investigating the effects of EXG-001. Via EXG-001, Zinc Finger and SCAN Domain Containing 4 (ZSCAN4) is brought to expression. ZSCAN4 regulates, amongst other things, telomere length. EXG-001 was able to lengthen telomeres in CD34-positive cells. Whether the reinfused cells also achieve the desirable effects in patients remains to be elucidated [208].

For the remaining IBMFSs, no clinical trials have started thus far. However, several successful proof-of-principle studies have been performed. Using viral vectors, RPS19 expression was successfully restored in vitro and in DBAS mouse models [209,210]. In a CAMT mouse model, lentiviral induction of *MPL* resulted in thrombocytosis, suggesting that MPL expression should be limited to early hematopoietic progenitors [211]. Indeed, with its expression limited to hematopoietic stem cells and megakaryocytes, MPL expression restored the CAMT phenotype [212]. Additionally, CRISPR/Cas9 showed an ability to restore *MPL* mutations in vitro [213]. Lastly, for SCN, both viral vectors as CRISPR/Cas9 have been used to restore *ELANE* and *HAX1* mutations and resolve neutropenia [214,215,216,217]. In contrast, no proof-of-principle studies investigating the use of gene therapy for SDS have been performed yet. To conclude, state-of-the-art techniques such as gene insertion or genome editing could provide new (curative) treatment options in the future.

## 7. Conclusions and Future Perspectives

BMF in children is caused by a broad spectrum of disorders, including IBMFSs, (idiopathic) AA, and MDS. Although pediatric BMF forms an extremely heterogenous group, HSCT plays an essential role in the treatment strategy as it can cure BMF in most patients, independent of the underlying cause. Depending on the severity of the disease, donor availability, and a patient’s condition to receive chemotherapy, HSCT is provided as an upfront or rescue therapy. Although the use of an MSD remains the modality of choice, alternative (mis)matched donors are increasingly being used. This is mainly achieved by adjusting GVHD prophylaxis with PT-CY or alpha/beta depletion, which results in impressive GVHD free survival rates approaching those after MSD HSCT. Due to these improvements in alternative donor use, HSCT has become more widely available.

However, especially for IBMFSs, long-term results are often unsatisfactory due to extra-hematological effects and the high incidence of secondary cancers. In general, an early time point for HSCT is argued to be before a transformation to a malignant disease as well as the use of non-myeloablative, less toxic conditioning regimens. However, preemptive HSCT should not be guided solely by clonal hematopoiesis. In particular situations, such as *SAMD9* and *SAMD9L* mutation-driven BMF, clonal hematopoiesis is part of an escape mechanism to restore hematopoiesis. Long-term post-HSCT follow-ups and management remain essential.

In the era of molecular diagnostics, enabling the increased identification of a genetic cause for BMF, gene therapy will gain terrain as the preferred curative treatment modality in the near future. Clinical management of pediatric patients with BMF remains challenging, and individualized treatment and surveillance plans are essential.

## Figures and Tables

**Table 1:** Overview of main characteristics of and treatment modalities for severe pediatric BMF.

Disorder	Main Hematological Manifestation	Drug Based Treatment	Cellular Therapy
Fanconi anemia	Pancytopenia	Supportive care, androgens	HSCT, gene therapy trials ongoing (NCT01331018, NCT03351868, NCT00272857, NCT03157804, NCT04069533, NCT00001399)
Telomere biology disorders (mainly dyskeratosis congenita)	Pancytopenia	Androgen therapy, e.g., danazol ^1^	HSCT, gene therapy at single case level (NCT04211714)
Diamond–Blackfan anemia syndrome	Anemia	<1 year: transfusions >1 year: + chelation therapy, steroids, e.g., prednisone	HSCT, gene therapy trials expected soon
Schwachman–Diamond syndrome	Neutropenia	Supportive care, G-CSF	HSCT
Severe congenital neutropenia	Neutropenia	Supportive care, G-CSF	HSCT
Congenital amegakaryocytic thrombocytopenia	Thrombocytopenia	-	HSCT
Thrombocytopenia absent radii	Thrombocytopenia	Platelet transfusions Platelet-stimulating agents, e.g., romiplostim and oprelvekin	HSCT
Aplastic anemia	Pancytopenia	IST + Epag	HSCT
Childhood MDS with low blasts	Multilineage dysplasia	IST	HSCT

^1^ Mainly studied in DC-associated pulmonary fibrosis in adult patients, but clinical trials are ongoing investigating broader application of androgens in TBDs including pediatric patients. HSCT, hematological stem cell transplantation; G-CSF, granulocyte colony-stimulating factor; IST, immunosuppressive therapy; Epag, Eltrombopag.

## Data Availability

Not applicable.
